# Microscopic Detection of *Entamoeba gingivalis* and *Trichomonas tenax* in Saliva and Subgingival Plaque Across Periodontal Health, Gingivitis, and Periodontitis: A Cross-Sectional Study

**DOI:** 10.3390/jcm15176665

**Published:** 2026-08-28

**Authors:** Bilge Meraci Yildiran, Mücahit Çakmak, Nuray Ercan

**Affiliations:** 1 Department of Periodontology, Faculty of Dentistry, Bolu Abant Izzet Baysal University, Bolu 14030, Türkiye; nuray.ercan@ibu.edu.tr; 2 Department of Parasitology, Faculty of Medicine, Bolu Abant Izzet Baysal University, Bolu 14030, Türkiye; mucahitcakmak@ibu.edu.tr

**Keywords:** *Entamoeba gingivalis*, gingivitis, periodontitis, *Trichomonas tenax*

## Abstract

**Background/Objectives**: The oral protozoa *Entamoeba gingivalis* (*E. gingivalis*) and *Trichomonas tenax* (*T. tenax*) have been implicated in periodontal disease, yet their relationship to clinical periodontal status remains unresolved. This study examined their detection frequency by light microscopy across periodontally healthy, gingivitis, and periodontitis groups, and its association with clinical periodontal parameters. **Methods**: In this single-center, cross-sectional study, 120 adults were allocated to three equal groups (*n* = 40 each): periodontally healthy, gingivitis, and periodontitis. From each participant, subgingival plaque was collected from the deepest periodontal pocket or sulcus of every quadrant, together with unstimulated saliva. Each specimen was examined as an unstained saline wet mount and with a Lugol iodine preparation under light microscopy. Data were analyzed using Chi-square, Kruskal–Wallis, and Mann–Whitney U tests. **Results**: *E. gingivalis* was detected in 20/120 participants: 7.5% in periodontally healthy, 20.0% in gingivitis, and 22.5% in periodontitis, a difference that did not reach statistical significance (*p* = 0.156). *T. tenax* was not detected in any participant (0/120). Positive participants had greater full-mouth probing pocket depth than negative participants (median 1.89 vs. 1.59 mm; *p* = 0.025). **Conclusions**: On microscopic examination of this sample, *E. gingivalis* detection did not differ significantly between periodontal groups, whereas *T. tenax* was not encountered. Among the clinical parameters examined, positivity for *E. gingivalis* was associated with probing pocket depth, consistent with the organism occupying the deep subgingival niche. Longitudinal molecular studies in larger, multicenter samples are warranted.

## 1. Introduction

Periodontal diseases are common chronic inflammatory conditions of the tooth-supporting tissues, of which gingivitis and periodontitis are the two principal forms [1]. Gingivitis is a reversible, plaque-induced inflammation confined to the gingiva, whereas in periodontitis, the inflammatory process extends to the supporting apparatus and causes irreversible clinical attachment and alveolar bone loss [2,3]. Periodontitis is now understood to be initiated not by select periopathogens acting alone but by a synergistic, dysbiotic microbial community in which low-abundance keystone species disrupt host–microbe homeostasis [4]. Within this framework, research has focused predominantly on the bacterial constituents of the subgingival biofilm, whereas its non-bacterial members, including protozoa, remain comparatively understudied. Two oral protozoa, *Entamoeba gingivalis (E. gingivalis*) and *Trichomonas tenax* (*T. tenax*), have been associated with periodontal disease [5,6], although pooled prevalence estimates across studies are markedly heterogeneous [5]. Their role nonetheless remains unresolved: whether they actively contribute to tissue destruction [7,8,9], opportunistically colonize an already dysbiotic and inflamed niche [10], or persist largely as commensal bystanders has not been established [11].

*Entamoeba gingivalis* was the first amoeba identified in dental plaque and resides in the gingival sulcus and periodontal pockets [9]. It occurs exclusively as a trophozoite of approximately 10–20 µm and, unlike the intestinal members of the genus, forms no cyst stage in vivo [12]. Because the trophozoite does not survive outside the host, transmission is thought to occur almost entirely through direct oral contact rather than through a food- or water-borne route [12]. Closely related to the enteric pathogen *Entamoeba histolytica*, it can invade the gingival epithelium and induce epithelial cell death by trogocytosis while inducing pro-inflammatory cytokine signaling [13]; yet despite this evidence of pathogenic potential, it is still widely regarded as a commensal, and its causal role in periodontal destruction remains contested [14].

*Trichomonas tenax* is a flagellated protozoan of roughly 6–10 µm that, like *E. gingivalis*, exists only as a trophozoite and forms no cyst; thus, its survival and transmission depend on the moist oral environment and direct contact [15]. It colonizes dental plaque and periodontal pockets and has also been reported in dental calculus and tonsillar crypts [16]. Although it is detected more frequently in individuals with poor oral hygiene and periodontal disease [16], the evidence for a pathogenic role is weaker and more contested than for *E. gingivalis*. A systematic review applying Koch’s postulates as revisited by Socransky concluded that the available evidence does not satisfy these postulates and is insufficient to establish a role for *T. tenax* in the etiopathogenesis of periodontitis [10].

Despite a growing number of prevalence reports, estimates for both protozoa vary widely across studies, reflecting differences in the populations screened and in the detection methods used [5,12,17]. Most prior studies have reported parasite positivity as group-level prevalence across health, gingivitis, and periodontitis [18,19,20,21,22], and the few that related positivity to clinical periodontal parameters relied largely on molecular detection [23,24]. Data linking direct microscopic detection of live trophozoites of both protozoa to clinical periodontal status, together with relevant covariates, therefore remain limited. Establishing whether the occurrence of these protozoa corresponds to clinical measures of periodontal status, rather than with broad diagnostic categories alone, would help clarify their relationship to disease. The primary objective of this cross-sectional study was to determine the detection frequency of *E. gingivalis* and *T. tenax* by light microscopy in saliva and subgingival plaque from periodontally healthy, gingivitis, and periodontitis groups. The secondary objective was to assess the association of protozoan detection with periodontal diagnosis and with clinical periodontal parameters. We hypothesized that *E. gingivalis* and *T. tenax* would be detected more frequently in participants with gingivitis and with periodontitis than in periodontally healthy participants.

## 2. Materials and Methods

### 2.1. Study Design

In this single-center, cross-sectional observational study, clinical periodontal examination and sampling were performed at the Department of Periodontology, Faculty of Dentistry, and parasitological analyses were performed at the Department of Parasitology, Faculty of Medicine, Bolu Abant Izzet Baysal University. Participants were enrolled between 22 December 2025 and 17 June 2026. Enrollment was consecutive within each diagnostic group, up to a predefined group size, as described below. The study was conducted in accordance with the Declaration of Helsinki and approved by the Bolu Abant Izzet Baysal University Non-Interventional Clinical Research Ethics Committee (Decision No: 2025/455, Date: 21 October 2025). Written informed consent was obtained from all participants before enrollment. The study was registered at ClinicalTrials.gov (NCT07551115).

### 2.2. Study Population

Eligible participants were adults presenting to the Department of Periodontology who consented to periodontal examination. Inclusion criteria were: age ≥ 18 years; presence of at least 10 natural teeth, with at least one tooth present in each of the four quadrants; and willingness to undergo examination and provide written informed consent. The threshold of 10 teeth was set so that full-mouth indices could be calculated from a sufficient number of teeth to be meaningful, and the quadrant requirement was applied because subgingival plaque was sampled from the deepest periodontal pocket or sulcus of every quadrant. Exclusion criteria were: systemic antibiotic use within the preceding 3 months; use of chlorhexidine or other antiseptic mouthwash within the preceding month; periodontal treatment within the preceding 3 months; immunosuppressive therapy or severe systemic immunodeficiency; and pregnancy or lactation. No participant screened during the enrollment period presented with necrotizing periodontal disease.

**Figure 1:**
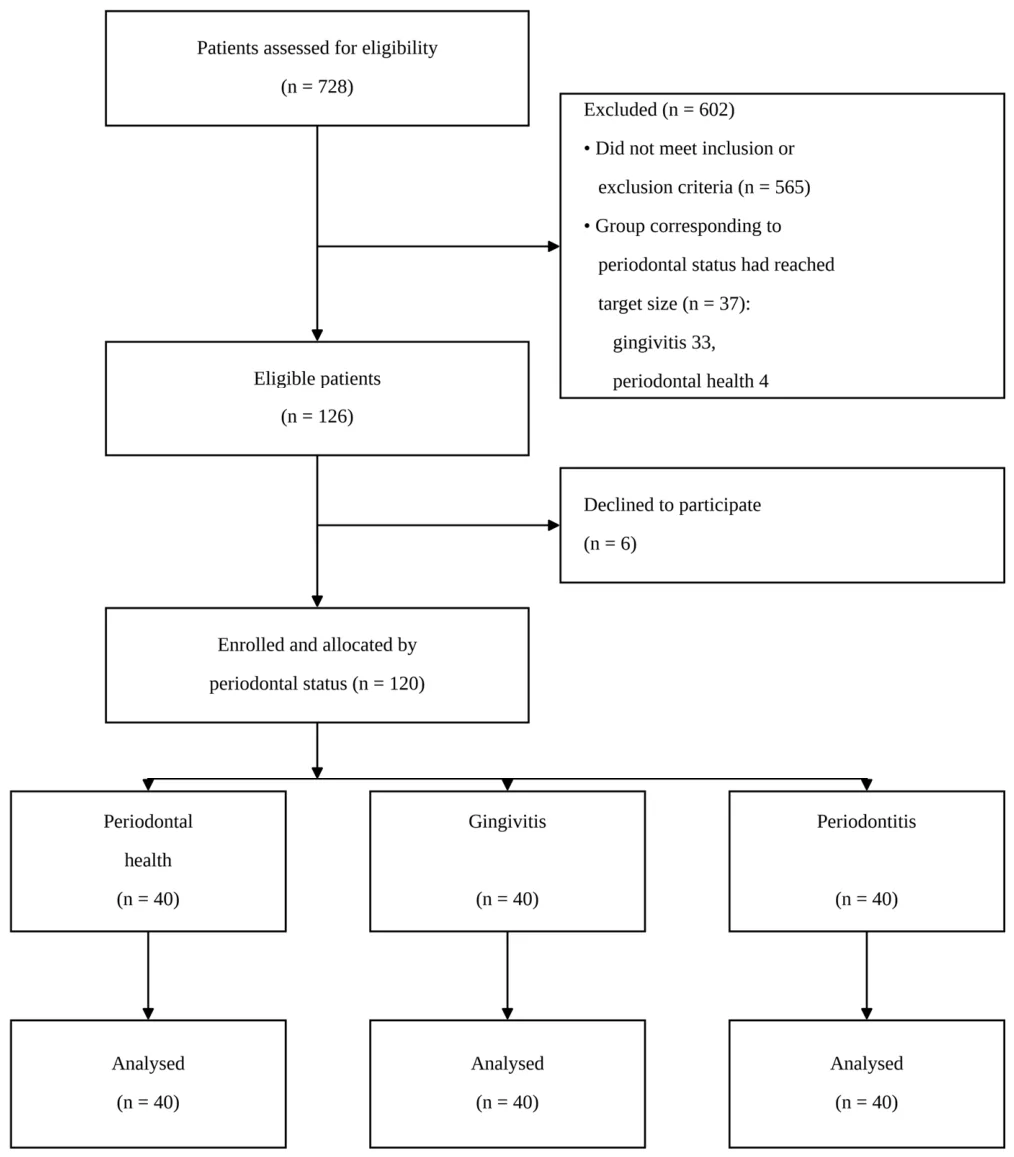
Flow of participants through screening, eligibility assessment, exclusion and allocation to the three periodontal groups, reported in accordance with STROBE.

During the enrollment period, 728 patients were assessed for eligibility. Of these, 565 did not meet the inclusion and exclusion criteria, and a further 37 were not enrolled because the group corresponding to their periodontal status had already reached its target size (33 gingivitis, 4 periodontal health), leaving 126 eligible patients. Of these, 6 declined to participate, resulting in 120 enrolled participants. Each eligible patient was assigned to the group corresponding to their periodontal status until that group reached its predefined size of 40. The flow of participants through screening, eligibility assessment, and group allocation is shown in Figure 1. No participant had missing data, and no enrolled participant was excluded from analysis.

Data on the following participant characteristics were obtained from a structured questionnaire at enrollment: age, sex, smoking status, presence of systemic disease, educational level, economic status, and oral hygiene habits.

An a priori power analysis was performed using G*Power software (version 3.1.9.7; Heinrich Heine University, Düsseldorf, Germany). The sample size calculation was based on a Chi-square test for comparing three independent groups. Assuming a medium effect size of Cohen’s w = 0.30, an alpha error probability of 0.05 and a statistical power of 80%, the minimum required total sample size was calculated as 108 participants. To ensure equal allocation among the three groups and to account for possible exclusions, 120 participants were included, with 40 participants in each group.

### 2.3. Periodontal Examination

Periodontal status was assessed by a single calibrated examiner (BMY) using a Williams periodontal probe (Hu-Friedy, Chicago, IL, USA), with findings recorded on a standardized chart. The plaque index (PI) and gingival index (GI) were recorded at four sites per tooth (buccal, lingual/palatal, mesial, distal), and bleeding on probing (BOP), probing pocket depth (PPD), and clinical attachment loss (CAL) at six sites per tooth (mesial, mid, and distal points of the buccal and palatal/lingual surfaces) [25,26]. BOP was expressed as the percentage of sites bleeding within 10 s of gentle probing; PPD was measured from the free gingival margin, and CAL from the cemento-enamel junction, to the base of the pocket. Full-mouth means were calculated for each index and measurement; in addition, a separate mean PPD (PPD-s) was calculated for the teeth from which plaque samples were obtained.

On the basis of these clinical measurements, together with radiographic assessment of alveolar bone loss using panoramic radiography, participants were assigned to one of three groups according to the 2017 World Workshop classification of periodontal and peri-implant diseases and conditions. Periodontal health was defined as BOP < 10%, probing depth ≤ 3 mm at all sites, and no clinical attachment loss or radiographic bone loss [2]. Gingivitis was defined as BOP ≥ 10% in the absence of clinical attachment loss and without radiographic bone loss [2,27]. Both groups therefore had an intact periodontium. Periodontitis was defined as detectable interdental CAL at ≥2 non-adjacent teeth, or buccal/oral CAL ≥ 3 mm with probing depth > 3 mm at ≥2 teeth, provided that the observed attachment loss could not be attributed to non-periodontal causes; for participants with periodontitis, stage and grade were additionally recorded following the same framework [3,28].

Reasons for tooth loss were determined from the participants’ history together with panoramic radiographic assessment. In the periodontally healthy and gingivitis groups, tooth loss was attributable to caries or trauma; in the periodontitis group, it was attributable to caries, trauma, or periodontitis.

### 2.4. Sample Collection

From each quadrant (maxillary right, maxillary left, mandibular right, and mandibular left), a subgingival plaque sample was collected from the tooth site exhibiting the deepest sulcus/pocket, yielding four separate plaque samples per participant. The target site was isolated from saliva with sterile cotton rolls, supragingival plaque was removed with sterile cotton pellets, and subgingival plaque was then collected from the sulcus/pocket with a sterile periodontal curette (Hu-Friedy, Chicago, IL, USA). Each plaque sample was placed in a separate tube containing 0.5 mL of 0.9% NaCl. Where no plaque was visible in the fluid after transfer, a further sample was taken from the next deepest periodontal pocket or sulcus in the same quadrant, so that the adequacy of sampling was assessed for every specimen before it was submitted for examination.

In addition, approximately 2 mL of unstimulated whole saliva was collected from each participant into a sterile tube; saliva was sampled at least 30 min after the participant had last eaten, drunk, or rinsed their mouth. All plaque and saliva samples were transported to the Department of Parasitology in a light-proof container within 10 to 15 min of collection.

### 2.5. Microscopic Examination

**Figure 2:**
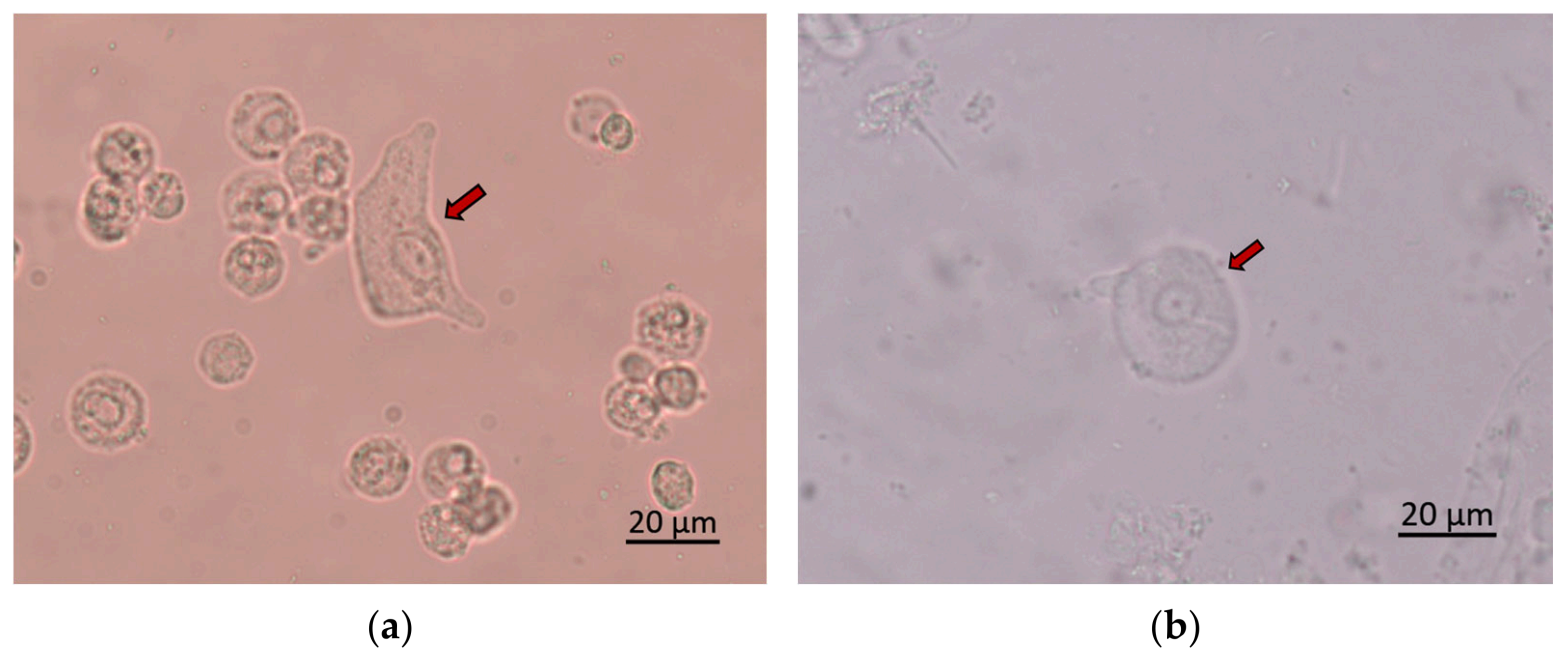
Light microscopy of *Entamoeba gingivalis (E. gingivalis*) trophozoites (×40; unstained saline preparation). (**a**) A trophozoite in a subgingival plaque specimen; (**b**) a trophozoite in a saliva specimen. Arrows indicate the *E. gingivalis* trophozoites. Scale bar = 20 µm.

All microscopic examinations were performed by an experienced parasitologist (MÇ) using Axio Scope A1 (ZEISS, Oberkochen, Germany). Prior to microscopy, the sample tubes were incubated (EFLAB EFLI-30 Incubator, Ankara, Türkiye) at 35–37 °C for 15–20 min to allow the contents to sediment. For each of the five specimens obtained from a participant (subgingival plaque from the maxillary right, maxillary left, mandibular left, and mandibular right quadrants, and saliva), one drop was withdrawn from the bottom of the tube with a Pasteur pipette and placed on a glass slide. A coverslip was held at a 45° angle and lowered gradually onto the droplet so that no air bubbles remained. For the unstained saline wet mount, low illumination was used to obtain contrast: the condenser was lowered and the diaphragm closed before examination was begun. Each preparation was scanned using a zigzag (serpentine) pattern so that no area of the coverslip was left unexamined, at 20× and 40× magnification as required (Figure 2a,b). For each specimen, a secondary preparation was then made with Lugol’s iodine solution (Merck, Darmstadt, Germany) and examined in the same manner. Photographs of the parasites were taken with a camera attached to the microscope (Axiocam 503 color, ZEISS), and their sizes were measured using the ZEN 2.3 software provided by the microscope manufacturer.

Where a structure could not be identified with confidence, it was not recorded as a protozoon. A specimen was recorded as positive when at least one organism was detected. Where no organism was seen in the initial preparation, a further preparation was made from a separate drop of the same specimen and examined again.

The parasitologist who performed the microscopic examination was blinded to the participants’ periodontal diagnosis. Microscopic examination was carried out after all periodontal measurements had been completed, so that the clinical assessment could not be influenced by the parasitological findings. The primary outcome was the presence of each oral protozoan, *E. gingivalis* and *T. tenax*, defined at the participant level as a binary variable: a participant was classified as positive for a given species if that species was detected in any of the collected specimens (subgingival plaque from any of the four quadrants or saliva), and negative otherwise. Because detection frequency was itself a primary outcome, a species not observed in any specimen was recorded as a detection frequency of zero and treated as a study result rather than as missing data.

### 2.6. Statistical Analysis

Statistical analyses were performed using IBM SPSS Statistics (v22.0; IBM Corp., Armonk, NY, USA). The conformity of continuous variables to a normal distribution was assessed by visual (histogram) and analytical (Kolmogorov–Smirnov/Shapiro–Wilk) methods. Because *T. tenax* was not detected in any participant, comparative analyses of protozoan presence could be performed only for *E. gingivalis*; the frequency of *T. tenax* is reported descriptively. Associations between the presence of *E. gingivalis* and the patient groups, and between questionnaire variables and the patient groups, were assessed using the Chi-square test. Clinical periodontal parameters (PI, GI, BOP, PPD, and CAL) were compared among the three periodontal groups using the Kruskal–Wallis test; pairwise comparisons between groups were performed using the Mann–Whitney U test with Bonferroni correction. Differences in clinical measurement values according to the presence or absence of *E. gingivalis*, overall and within each group, were analyzed using the Mann–Whitney U test. A total type I error level of 5% was used to determine statistical significance.

## 3. Results

A total of 120 participants were included, evenly allocated to the healthy, gingivitis, and periodontitis groups (Figure 1). Across the three groups (*n* = 40 each), all clinical periodontal parameters increased stepwise from health to periodontitis (*p* < 0.01). PI, GI, PPD, and PPD-s differed significantly among all three groups, whereas BOP separated the healthy group from both the gingivitis and periodontitis groups, which did not differ from each other. Attachment loss was confined to the periodontitis group (median CAL 3.74 [2.48–5.96]; 0 in the other groups by case definition). The periodontitis group was also older (median 47.50 years, range 34–68) than the healthy and gingivitis groups, which were comparable in age (Table 1).

*E. gingivalis* was detected in 20 of the 120 participants. Patient-level detection was numerically higher in the gingivitis (8/40, 20.0%) and periodontitis (9/40, 22.5%) groups than in the healthy group (3/40, 7.5%), but this difference was not statistically significant (*p* = 0.156). Within the periodontitis group, detection did not differ significantly across stages II–IV (*p* = 0.232). *T. tenax* was not detected in any participant (0/120) in any group using the microscopic protocol employed, and no comparison was therefore performed (Table 2).

*E. gingivalis* positivity was not significantly associated with any of the demographic, socioeconomic, systemic, or oral-hygiene variables examined (*p* > 0.05). The lowest *p*-value was observed for education level (*p* = 0.075), which did not reach statistical significance; positivity ranged from 0% (middle school, associate degree) to 33.3% (primary school) across educational categories. Detection frequencies by sex, smoking status, oral-hygiene behaviors, and the remaining variables are presented in Table 3.

Among the variables examined, only full-mouth PPD differed significantly between the two groups: *E. gingivalis*-positive participants had greater probing depth than negative participants (*p* = 0.025). PPD at the sampled sites was likewise higher in positive participants but reached only borderline significance (*p* = 0.050). Body mass index (BMI), age, number of remaining teeth, PI, GI, BOP, and CAL did not differ significantly between the positive and negative participants (*p* > 0.05; Table 4).

## 4. Discussion

In this single-center cross-sectional study, in which both protozoa were sought by light microscopy, *E. gingivalis* was detected in 20 of the 120 participants, whereas *T. tenax* was not encountered. Detection of *E. gingivalis* did not differ significantly between the three periodontal groups. Among the clinical parameters examined, *E. gingivalis* detection was associated with probing pocket depth, consistent with the organism occupying the deep subgingival niche that pocket depth reflects.

Because the three periodontal groups were enrolled in equal numbers by design, *E. gingivalis* detection is best considered by group rather than as a single overall proportion, and the group-level values represent detection frequencies within a quota-sampled series rather than prevalence estimates. It was detected in 7.5% of the healthy group, 20.0% in gingivitis, and 22.5% in periodontitis, substantially lower than the pooled estimates of a recent meta-analysis of 20 studies, which reported approximately 45% in gingivitis and 55% in periodontitis [5]. These pooled values are difficult to interpret in isolation. The contributing estimates were highly heterogeneous, with prevalence ranging from 15% to 86% in gingivitis and from 14% to 100% in periodontitis, and between-study heterogeneity above 90% [5]. One source of this dispersion is the detection method. Because molecular assays are more sensitive, they may yield considerably higher frequencies [5]; a nested-PCR survey, for example, detected *E. gingivalis* in 54.3% of periodontally healthy individuals [17]. Molecular detection does not invariably produce this result, however: a nested-PCR study using the same three-group design reported an overall frequency of 17.6%, with 7.5% in periodontal health, 17.0% in gingivitis and 28.3% in periodontitis [29], closely comparable to the frequencies observed here.

The population sampled appears to be a stronger determinant of absolute frequency than the detection method. The first molecular study of Turkish individuals detected *E. gingivalis* by PCR in only 20.2% overall: 30.5% in those with dental disease and 13.8% in healthy controls [21]. Both this study and the Mexican nested-PCR survey used molecular detection, yet their prevalence estimates differ markedly, indicating that the population sampled is a dominant determinant of the absolute frequency. Turkish direct-microscopy studies span a wide range, with lower estimates of 19.4% [18] and 21.8% [30] alongside considerably higher yields of 44.2% overall, reaching 80% in periodontitis [20], and 54.3% in a sample of 46 patients restricted to periodontal disease [31]. Direct comparison with the present study is limited principally because identification in these reports rested on the morphology of fixed organisms in Trichrome- or Giemsa-stained smears [18,31], whereas detection here rested on the motility of live trophozoites in unstained wet mounts; the two approaches define positivity differently rather than differing only in yield. Consistent with this, one Turkish study that applied both approaches to the same participants obtained fewer positive results by staining than by immediate direct examination [30]. Two further differences bear on comparability: most of these values are overall rates from mixed clinical samples rather than group-specific rates, and one included no periodontally healthy group, so its overall value describes a disease-only sample [31]. Acıoz et al. also examined three groups of 40 by light microscopy [20]; differences in the anatomical sites sampled, in sampling procedure and in participant selection, including exclusion criteria, may have contributed to the divergent findings of the two studies. Our group-specific frequencies lie at the lower end of a wide regional range whose dispersion reflects differences in case definition, sampling and participant selection rather than detection method alone.

The two detection approaches also differ in what they measure, and not only in analytical sensitivity. Light microscopy of an unstained wet mount detects motile trophozoites, whereas PCR does not discriminate between viable organisms, non-viable organisms and free DNA. In a study of patients with diabetes mellitus that applied both methods to the same participants, microscopy and PCR yielded comparable overall positivity for the two organisms combined (27.2% and 29.2%) [32]. PCR is also not free of false-negative results: in the study that established its use for *E. gingivalis* in periodontal pockets, 12 specimens positive by both clinical assessment and microscopy were negative by PCR, which the authors attributed to heterogeneity of the sampled material, to genetic variability within the species, and to the possible presence of other Entamoeba species in the sulcus [33]. The constraints of the two methods are therefore distinct rather than nested. Microscopy was selected here as the most rapid and least costly method for characterizing the distribution of these organisms across the three diagnostic groups within a defined recruitment period, and because it detects the viable trophozoite, which is the phenotype of interest when colonization of the subgingival niche is in question. Because the two approaches interrogate different targets, combining them in the same samples would exploit their complementary strengths, microscopy confirming a viable trophozoite and PCR adding species-level sensitivity, and would clarify how far published estimates diverge for methodological reasons.

Within our sample, *E. gingivalis* detection was associated with probing pocket depth. Positive participants had greater full-mouth probing depth than negative participants, and probing depth at the sampled sites followed the same direction. This finding is in agreement with molecular data from a Chinese cross-sectional study, in which the relative abundance of *E. gingivalis* determined by rRNA gene sequencing correlated with probing depth more strongly than the red-complex pathogens assessed concurrently. Amoebic abundance also increased in parallel with gingival crevicular fluid levels of interleukin-1β (IL-1β), IL-8 and tumor necrosis factor-α (TNF-α) [24]. A larger PCR-based study in Jordan reported a strong association between *E. gingivalis* and the presence of periodontal disease, although its association with more specific disease severity categories did not reach significance [22]. Clinical attachment loss did not differentiate positive from negative participants in the present sample, and its reported relationship with the parasite is inconsistent, ranging from a positive association in one molecular study [24] to an inverse association in a microscopy-based study [34]. In the present study, detection was associated with probing pocket depth, whereas no significant difference was observed between the periodontally healthy, gingivitis, and periodontitis groups. The greater probing depth observed in *E. gingivalis*-positive participants is biologically plausible, as pocket depth reflects the subgingival niche this organism occupies. *E. gingivalis* resides in the gingival sulcus and periodontal pocket, where the protected, low-oxygen environment favors its survival [35]. Within this niche, the trophozoite ingests fragments of viable host cells through trogocytosis, invades the oral mucosa, induces epithelial cell death, and elicits a pro-inflammatory response involving TNF, IL-8, and the chemokines C-C motif chemokine ligand 20 (CCL20) and C-X-C motif chemokine ligand 2 (CXCL2) [13]. Consistent with these experimental findings, the crevicular-fluid cytokines that accompany higher amoebic abundance in vivo mirror the same response [24]. This ecological preference may therefore underlie the association observed in the present study. Whether the organism modifies this niche or merely occupies it has been addressed in a prospective interventional study using PCR-based detection. Following scaling and root planing, clinical parameters improved and the bacterial load declined; however, only *T. tenax* was significantly reduced, and the change in *E. gingivalis* was not associated with the improvement in probing depth [23]. These observations are more consistent with *E. gingivalis* colonizing the deep, inflamed pocket than with its contributing to the formation of that depth. This interpretation is congruent with the cross-sectional association reported here and is further supported by its agreement with independent molecular data.

*T. tenax* was not detected in any of the 120 participants, in either the unstained or the Lugol preparation. This protozoan is identified primarily by its characteristic motility in unstained saline wet mounts [12], and motility declines progressively once the specimen leaves the host [12,16]. When the organism is scarce, has become non-motile prior to examination, or lies outside the examined field, it may be overlooked, and iodine staining does not reliably reveal it. Several features of the protocol were intended to reduce the likelihood of such false-negative results: subgingival plaque from each quadrant and unstimulated saliva were sampled; specimens were held in the saline tube rather than on a glass slide, with a slide prepared only at the time of reading [12,16]; each preparation was scanned in a zigzag pattern so that no area of the coverslip was left unexamined; each specimen was examined both as an unstained wet mount and with a Lugol preparation; and where no organism was seen initially, a further preparation was made from a separate drop. A negative result therefore did not rest on a single mounted field. *T. tenax* nonetheless remained undetected in all specimens.

*T. tenax* is consistently the less prevalent of the two oral protozoa [5]. Reported detection rates in Turkish populations examined by microscopy are low, at 2.2% [18], 4.5% [30] and 5.8% [20]; a further Turkish study reported no positive cases [31]. In the study reporting 4.5%, the organism was identified alone in 1% of 220 participants and together with *E. gingivalis* in a further 3.6%, and its relationship to periodontal disease was described as less evident than that of *E. gingivalis* [30]. Higher prevalences are reported mainly from molecular studies and other populations [5]. A Jordanian PCR study reported 25.6% in periodontitis and 12.2% overall [22], comparable to the pooled meta-analytic estimates of 14% in gingivitis and 26% in periodontitis [5]. Molecular detection does not invariably yield high frequencies. Indeed, a PCR-based study of patients with periodontal disease detected *T. tenax* in 13.5% of participants, almost entirely in periodontitis [36]. Where detected, *T. tenax* has been associated with periodontitis severity [34] and has been shown to decrease following non-surgical periodontal therapy [23,34]. The absence of detection in the present study is compatible with the low prevalences previously reported for *T. tenax*, particularly in Turkish populations. It cannot, however, be attributed to prevalence alone, since the sensitivity of the detection method also bears on a negative result. It is presented as an observation in this sample with the protocol described, rather than as evidence that the organism is absent from this population.

The biological significance of *E. gingivalis* detection remains unsettled. Although long regarded as a harmless commensal, the organism is closely related to the pathogen *E. histolytica* and is markedly enriched in periodontitis, and its status has been proposed for reconsideration as a potential contributor to the disease [9]. It ingests host cells, including the neutrophils that constitute the first line of innate defense, thereby potentially impairing their protective function within the pocket. The deep, inflamed pocket may favor colonization; alternatively, the organism may aggravate an established lesion, or both may arise from the underlying bacterial dysbiosis [9].

Its potential utility as a diagnostic marker or therapeutic target is similarly uncertain. Reported prevalences vary excessively across populations and detection methods for the organism to serve as a reliable diagnostic indicator, and a causal role has not been established [5,9]. Non-surgical periodontal therapy reduces oral protozoa to varying degrees as it disrupts the subgingival biofilm [23,34]; however, the improvement in probing depth paralleled the reduction in bacterial load rather than the change in protozoa, and no benefit attributable specifically to elimination of the amoeba has been demonstrated [23]. On present evidence, the data more readily support interpreting *E. gingivalis* as a marker of the deep subgingival niche that pocket depth reflects than as an antiprotozoal target, although its potential contribution to periodontitis has been argued to merit reconsideration [9]. The present findings show an association between *E. gingivalis* detection and probing pocket depth, and remain compatible with, though do not establish, a contributory role.

Several limitations of this study should be acknowledged. The study was conducted at a single-center in a single population, so the findings may have limited generalizability. This constraint is particularly relevant for an organism whose reported detection frequency varies markedly between populations. Detection relied solely on light microscopy, which is less sensitive than PCR, particularly for *T. tenax*. The Lugol preparation mitigates but does not eliminate this limitation, and no molecular confirmation was undertaken. The zero detection rate for *T. tenax* may reflect a genuinely low frequency of the organism in the population examined, but false-negative results attributable to the sensitivity of the detection method cannot be excluded. Intra-examiner agreement was not assessed. The unstained wet mount is a temporary preparation of living material, so a second reading is not a repeated measurement of the same object. The cross-sectional design captures associations at a single time point, and therefore cannot establish temporal sequence or causality. These limitations are counterbalanced by several strengths. Diagnostic groups were defined by the 2017 World Workshop classification, giving three well-characterized groups of equal size. Subgingival plaque was collected from the deepest periodontal pocket or sulcus in each quadrant, the site most likely to harbor these organisms, and unstimulated saliva was sampled in addition. Every specimen was examined separately, both as an unstained saline wet mount and with a Lugol secondary preparation. Combining light microscopy with molecular detection in the same specimens is planned for subsequent studies, so that the viability information provided by direct microscopy and the species-level confirmation provided by PCR can be obtained together.

## 5. Conclusions

Within this single-center sample examined by light microscopy, *E. gingivalis* was detected in all three periodontal groups, with no significant difference between them, whereas *T. tenax* was not encountered. Among the clinical parameters examined, *E. gingivalis* detection was associated with probing pocket depth. This is consistent with the organism occupying the deep subgingival niche that pocket depth reflects, rather than denoting a specific disease stage. On present evidence, *E. gingivalis* is more appropriately interpreted as a marker of that niche, and whether it also contributes to periodontal tissue breakdown remains to be established. Longitudinal, molecular investigations in larger, multicenter samples are required to distinguish these possibilities, and to determine whether the organism has clinical value as a diagnostic indicator or therapeutic target.

## Figures and Tables

**Table 1:** Age, number of remaining teeth, and full-mouth clinical periodontal parameters across the three study groups.

Variables	Groups	*n*	Median (Min–Max)	*p*-Value
Age	Healthy	40	33.50 (22–47) ^a^	<0.01 *
	Gingivitis	40	35.50 (18–50) ^a^	
	Periodontitis	40	47.50 (34–68) ^b^	
Number of remaining teeth	Healthy	40	28 (17–28) ^a^	<0.01 *
	Gingivitis	40	27 (18–28) ^b^	
	Periodontitis	40	22 (11–28) ^c^	
PI	Healthy	40	0.41 (0.18–1.20) ^a^	<0.01 *
	Gingivitis	40	1.08 (0.36–2.90) ^b^	
	Periodontitis	40	1.35 (0.76–2.67) ^c^	
GI	Healthy	40	0.17 (0.04–0.63) ^a^	<0.01 *
	Gingivitis	40	1.24 (0.77–2.00) ^b^	
	Periodontitis	40	1.42 (0.85–2.33) ^c^	
BOP (%)	Healthy	40	4.58 (1.19–8.33) ^a^	<0.01 *
	Gingivitis	40	31.55 (15.38–78.19) ^b^	
	Periodontitis	40	33.33 (12.82–93.59) ^b^	
PPD	Healthy	40	1.47 (1.04–1.89) ^a^	<0.01 *
	Gingivitis	40	1.60 (1.16–2.54) ^b^	
	Periodontitis	40	2.66 (1.43–4.47) ^c^	
PPD-s	Healthy	40	2.25 (1.75–3.00) ^a^	<0.01 *
	Gingivitis	40	2.75 (2.00–3.00) ^b^	
	Periodontitis	40	4.62 (3.50–9.00) ^c^	
CAL	Healthy	40	0.00 ^a^	<0.01 *
	Gingivitis	40	0.00 ^a^	
	Periodontitis	40	3.74 (2.48–5.96) ^b^	

Values are expressed as median (min–max) (*n* = 40 per group). Omnibus differences among the three groups were assessed with the Kruskal–Wallis test (* *p* < 0.01); pairwise comparisons were performed with the Mann–Whitney U test with Bonferroni correction. Superscript letters: within each row, groups not sharing a common superscript letter (a, b, c) differ significantly (Bonferroni-corrected *p* < 0.05). PI, plaque index; GI, gingival index; BOP, bleeding on probing; PPD, probing pocket depth; PPD-s, probing pocket depth at the sampled sites; CAL, clinical attachment loss.

**Table 2:** Patient-level detection of *Entamoeba gingivalis* and *Trichomonas tenax* in subgingival plaque and saliva according to periodontal status.

Periodontal Status	*n*	*E. gingivalis* Positive		*T. tenax* Positive	
		*n* (%)	*p*-Value	*n* (%)	*p*-Value
Healthy	40	3 (7.5)	0.156	0	–
Gingivitis	40	8 (20.0)		0	
Periodontitis	40	9 (22.5)		0	
Stage II	14	1 (7.1)	0.232	0	–
Stage III	16	5 (31.3)		0	
Stage IV	10	3 (30.0)		0	

Data are presented as n (%) of positive patients; a participant was classified as positive when the organism was detected in any sampled specimen (subgingival plaque or saliva). Between-group comparisons were performed with the Chi-square test; a *p*-value < 0.05 was considered statistically significant. –, not calculable (no positive cases). *E. gingivalis*, *Entamoeba gingivalis*; *T. tenax*, *Trichomonas tenax*.

**Table 3:** Detection of *Entamoeba gingivalis* according to demographic characteristics, systemic health status, and oral hygiene practices.

Variables	Category	Total *n*	*E. gingivalis* Positive		*E. gingivalis* Negative		*p*-Value
			*n*	%	*n*	%	
Sex	Female	69	10	14.5	59	85.5	0.457
	Male	51	10	19.6	41	80.4	
Education level	Primary school	15	5	33.3	10	66.7	0.075
	Middle school	11	0	0.0	11	100.0	
	High school	31	5	16.1	26	83.9	
	Associate degree	9	0	0.0	9	100.0	
	Bachelor’s degree	37	9	24.3	28	75.7	
	Postgraduate	17	1	5.9	16	94.1	
Income (monthly)	Low	26	6	23.1	20	76.9	0.460
	Middle	38	7	18.4	31	81.6	
	High	56	7	12.5	49	87.5	
Smoking status	Never smoked	69	9	13.0	60	87.0	0.403
	Former smoker	17	3	17.6	14	82.4	
	Current smoker	34	8	23.5	26	76.5	
Alcohol consumption	No	106	17	16.0	89	84.0	0.611
	Yes	14	3	21.4	11	78.6	
Systemic disease	No	93	16	17.2	77	82.8	0.515
	Yes	27	4	14.8	23	85.2	
Toothbrushing frequency	Less than twice daily	56	9	16.1	47	83.9	0.986
	Twice daily	58	10	17.2	48	82.8	
	Three or more times daily	6	1	16.7	5	83.3	
Toothbrushing duration	<1 min	28	2	7.1	26	92.9	0.303
	1–2 min	66	13	19.7	53	80.3	
	>2 min	26	5	19.2	21	80.8	
Interdental cleaning	No	73	13	17.8	60	82.2	0.439
	Yes	47	7	14.9	40	85.1	
Tongue cleaning	No	85	12	14.1	73	85.9	0.243
	Yes	35	8	22.9	27	77.1	
Dental visit frequency	>12 months	46	11	23.9	35	76.1	0.244
	6–12 months	24	3	12.5	21	87.5	
	<6 months	50	6	12.0	44	88.0	
Prosthetic restoration	No	88	12	13.6	76	86.4	0.140
	Yes	32	8	25.0	24	75.0	
Tap water consumption	No	74	11	14.9	63	85.1	0.502
	Yes	46	9	19.6	37	80.4	

Data are presented as *n* (%); percentages are row-wise (the proportion of *E. gingivalis*-positive and -negative patients within each category). Associations were assessed with the Chi-square test; a *p*-value < 0.05 was considered statistically significant. *E. gingivalis*, *Entamoeba gingivalis*.

**Table 4:** Comparison of age, body mass index, and clinical periodontal parameters between *E. gingivalis*-positive and -negative participants.

Variables	Groups	*n*	Median (Min–Max)	*p*-Value
BMI	*E. gingivalis* negative	100	25.42 (19.04–38.86)	0.510
	*E. gingivalis* positive	20	25.80 (20.76–32.05)	
Age	*E. gingivalis* negative	100	39 (18–68)	0.333
	*E. gingivalis* positive	20	45.50 (20–61)	
Number of remaining teeth	*E. gingivalis* negative	100	27 (11–28)	0.125
	*E. gingivalis* positive	20	25 (13–28)	
PI	*E. gingivalis* negative	100	1.00 (0.18–2.90)	0.224
	*E. gingivalis* positive	20	1.03 (0.42–2.67)	
GI	*E. gingivalis* negative	100	1.13 (0.04–2.00)	0.261
	*E. gingivalis* positive	20	1.16 (0.27–2.33)	
BOP (%)	*E. gingivalis* negative	100	27.66 (1.19–89.74)	0.275
	*E. gingivalis* positive	20	27.98 (2.98–93.59)	
PPD	*E. gingivalis* negative	100	1.59 (1.04–4.47)	0.025 *
	*E. gingivalis* positive	20	1.89 (1.29–3.99)	
PPD-s	*E. gingivalis* negative	100	2.50 (1.75–9.00)	0.050
	*E. gingivalis* positive	20	3.00 (2.00–7.50)	
CAL	*E. gingivalis* negative	100	0.00 (0.00–5.90)	0.179
	*E. gingivalis* positive	20	0.00 (0.00–5.96)	

Values are expressed as median (min–max). The *E. gingivalis*-positive and -negative groups were compared with the Mann–Whitney U test; * *p* < 0.05. BMI, body mass index; PI, plaque index; GI, gingival index; BOP, bleeding on probing; PPD, probing pocket depth; PPD-s, probing pocket depth at the sampled sites; CAL, clinical attachment loss.

## Data Availability

The data are available from the corresponding author upon reasonable request due to privacy restrictions.

## Abbreviations

The following abbreviations are used in this manuscript:

BMI	Body Mass Index
BOP	Bleeding on Probing
CAL	Clinical Attachment Loss
CCL20	C-C Motif Chemokine Ligand 20
CXCL2	C-X-C Motif Chemokine Ligand 2
GI	Gingival Index
IL-1β	Interleukin-1β
IL-8	Interleukin-8
PCR	Polymerase Chain Reaction
PI	Plaque Index
PPD	Probing Pocket Depth
PPD-s	Probing Pocket Depth at sampled sites
TNF-α	Tumor Necrosis Factor-α

## References

[1] Caton J.G., Armitage G., Berglundh T., Chapple I.L.C., Jepsen S., Kornman K.S., Mealey B.L., Papapanou P.N., Sanz M., Tonetti M.S. (2018). A new classification scheme for periodontal and peri-implant diseases and conditions–Introduction and key changes from the 1999 classification. J. Clin. Periodontol..

[2] Chapple I.L.C., Mealey B.L., Van Dyke T.E., Bartold P.M., Dommisch H., Eickholz P., Geisinger M.L., Genco R.J., Glogauer M., Goldstein M. (2018). Periodontal health and gingival diseases and conditions on an intact and a reduced periodontium: Consensus report of workgroup 1 of the 2017 World Workshop on the Classification of Periodontal and Peri-Implant Diseases and Conditions. J. Clin. Periodontol..

[3] Papapanou P.N., Sanz M., Buduneli N., Dietrich T., Feres M., Fine D.H., Flemmig T.F., Garcia R., Giannobile W.V., Graziani F. (2018). Periodontitis: Consensus report of workgroup 2 of the 2017 World Workshop on the Classification of Periodontal and Peri-Implant Diseases and Conditions. J. Clin. Periodontol..

[4] Hajishengallis G., Lamont R.J. (2012). Beyond the red complex and into more complexity: The polymicrobial synergy and dysbiosis (PSD) model of periodontal disease etiology. Mol. Oral Microbiol..

[5] Moosazadeh M., Sabeti M.A., Hashemi S.M., Ghazalgoo A., Mousavi T., Mahdavi S., Ghadirzadeh E. (2025). The Relationship Between *Entamoeba Gingivalis* and *Trichomonas Tenax* with Periodontitis and Gingivitis: A Systematic Review, Meta-Analysis, and Meta-Regression. J. Evid.-Based Dent. Pract..

[6] Badri M., Olfatifar M., Abdoli A., Houshmand E., Zarabadipour M., Abadi P.A., Johkool M.G., Ghorbani A., Eslahi A.V. (2021). Current Global Status and the Epidemiology of *Entamoeba gingivalis* in Humans: A Systematic Review and Meta-analysis. Acta Parasitol..

[7] Bao X., Wiehe R., Dommisch H., Schaefer A.S. (2020). *Entamoeba gingivalis* Causes Oral Inflammation and Tissue Destruction. J. Dent. Res..

[8] Hong Z.-B., Lai Y.-T., Chen C.-H., Chen Y.-J., Chen C.-C., Lin W.-C. (2023). *Trichomonas tenax* induces barrier defects and modulates the inflammatory cytotoxicity of gingival and pulmonary epithelial cells. Parasite.

[9] Bonner M., Fresno M., Gironès N., Guillén N., Santi-Rocca J. (2018). Reassessing the Role of *Entamoeba gingivalis* in Periodontitis. Front. Cell. Infect. Microbiol..

[10] Bisson C., Dridi S.M., Machouart M. (2019). Assessment of the role of Trichomonas tenax in the etiopathogenesis of human periodontitis: A systematic review. PLoS ONE.

[11] Stensvold C.R., Nielsen M., Baraka V., Lood R., Fuursted K., Nielsen H.V. (2021). *Entamoeba gingivalis*: Epidemiology, genetic diversity and association with oral microbiota signatures in North Eastern Tanzania. J. Oral Microbiol..

[12] Santos J.O., Roldán W.H. (2023). *Entamoeba gingivalis* and *Trichomonas tenax*: Protozoa parasites living in the mouth. Arch. Oral Biol..

[13] Bao X., Weiner J., Meckes O., Dommisch H., Schaefer A.S. (2021). *Entamoeba gingivalis* Exerts Severe Pathogenic Effects on the Oral Mucosa. J. Dent. Res..

[14] Becker C., Adam A., Dommisch H., Stach T., Schaefer A.S. (2023). In vitro induction of *Entamoeba gingivalis* cyst-like structures from trophozoites in response to antibiotic treatment. Front. Cell. Infect. Microbiol..

[15] Matthew M., Ketzis J., Mukaratirwa S., Yao C. (2025). Transmission Dynamics of *Trichomonas tenax*: Host and Site Specificity, Zoonotic Potential, and Environmental Factors. Microorganisms.

[16] Matthew M.A., Yang N., Ketzis J., Mukaratirwa S., Yao C. (2023). *Trichomonas tenax*: A Neglected Protozoan Infection in the Oral Cavities of Humans and Dogs—A Scoping Review. Trop. Med. Infect. Dis..

[17] Garcia G., Ramos F., Maldonado J., Fernandez A., Yáñez J., Hernandez L., Gaytán P. (2018). Prevalence of two *Entamoeba gingivalis* ST1 and ST2-kamaktli subtypes in the human oral cavity under various conditions. Parasitol. Res..

[18] Abualqomsaan M., Özensoy Töz S., Yolasiğmaz A., Turgay N. (2010). Periodontal Hastalığı Bulunan Kişilerde Diş Eti Plaklarında *Entamoeba gingivalis* ve *Trichomonas tenax* Araştırılması. Turk. J. Parasitol..

[19] Yazar S., Cetinkaya U., Hamamci B., Alkan A., Sisman Y., Esen C., Kolay M. (2016). Investigation of *Entamoeba gingivalis* and *Trichomonas tenax* in Periodontitis or Gingivitis Patients in Kayseri. Turk. J. Parasitol..

[20] Aciöz M., Ak G., Bozkaya F. (2025). Investigation of *Entamoeba gingivalis* and *Trichomonas tenax* in gingivitis and periodontitis patients. BMC Oral Health.

[21] Örsten S., Šahin C., Yllmaz E., Akyön Y. (2023). First molecular detection of *Entamoeba gingivalis* subtypes in individuals from Turkey. Pathog. Dis..

[22] Yaseen A., Mahafzah A., Dababseh D., Taim D., Hamdan A.A., Al-Fraihat E., Hassona Y., Şahin G.Ö., Santi-Rocca J., Sallam M. (2021). Oral Colonization by *Entamoeba gingivalis* and *Trichomonas tenax*: A PCR-Based Study in Health, Gingivitis, and Periodontitis. Front. Cell. Infect. Microbiol..

[23] Dubar M., Zaffino M.L., Remen T., Thilly N., Cunat L., Machouart M.C., Bisson C. (2020). Protozoans in subgingival biofilm: Clinical and bacterial associated factors and impact of scaling and root planing treatment. J. Oral Microbiol..

[24] Jiao J., Bie M., Xu X., Duan D., Li Y., Wu Y., Zhao L. (2022). *Entamoeba gingivalis* is associated with periodontal conditions in Chinese young patients: A cross-sectional study. Front. Cell. Infect. Microbiol..

[25] Löe H., Silness J. (1963). Periodontal disease in pregnancy I. Prevalence and severity. Acta Odontol. Scand..

[26] Löe H. (1967). The Gingival Index, the Plaque Index and the Retention Index Systems. J. Periodontol..

[27] Trombelli L., Farina R., Silva C.O., Tatakis D.N. (2018). Plaque-induced gingivitis: Case definition and diagnostic considerations. J. Periodontol..

[28] Tonetti M.S., Greenwell H., Kornman K.S. (2018). Staging and grading of periodontitis: Framework and proposal of a new classification and case definition. J. Periodontol..

[29] Tahmasebi E., Ghanimatdan M., Hajisadeghi S., Rafiei E., Sharifi Y., Jafari Nodoushan Z., Khedmat L., Rahimi M. (2025). A Case Control Study to Assess Frequency, Risk Factors and Genetic Diversity of *Entamoeba gingivalis*. Oral Dis..

[30] Özçelik S., Gedik T., Gedik R., Malatyali E. (2010). Ağız ve Diş Sağlığı ile *Entamoeba gingivalis* ve *Trichomonas tenax* Varlığı Arasındaki İlişkinin Araştırılması [Investigation of The Relationship Between Oral and Dental Health and Presence of *Entamoeba gingivalis* and *Trichomonas tenax*]. Turk. J. Parasitol..

[31] Özdemir Öğrü B., Yildirim M.Z., Köse O. (2022). Burdur Ağız ve Diş Sağlığı Merkezi’nde Periodontal Bulguları Olan Hastalarda *Entamoeba Gingivalis* ve *Trichomonas Tenax’ın* Araştırılması. Süleyman Demirel Üniversitesi Sağlık Bilim. Derg..

[32] Masoori L., Baharvand P., Khalaf A.K., Selahbarzin B., Sakifar F., Mahmoudvand H. (2025). Frequency, socio-economic characteristics, and risk factors of oral cavity parasites in diabetes mellitus patients from Lorestan Province, Iran; a case-control study. Front. Cell. Infect. Microbiol..

[33] Bonner M., Amard V., Bar-Pinatel C., Charpentier F., Chatard J.-M., Desmuyck Y., Ihler S., Rochet J.-P., de La Tribouille V.R., Saladin L. (2014). Detection of the amoeba *Entamoeba gingivalis* in periodontal pockets. Parasite.

[34] Rashidi Maybodi F., Haerian Ardakani A., Fattahi Bafghi A., Haerian Ardakani A., Zafarbakhsh A. (2016). The Effect of Nonsurgical Periodontal Therapy on *Trichomonas Tenax* and *Entamoeba Gingivalis* in Patients with Chronic Periodontitis. J. Dent..

[35] Fadhil Ali Malaa S., Abd Ali Abd Aun Jwad B., Khalis Al-Masoudi H. (2022). Assessment of *Entamoeba Gingivalis* and *Trichomonas Tenax* in Patients with Chronic Diseases and its Correlation with Some Risk Factors. Arch. Razi Inst..

[36] Chitpitaklert P., Boonsuya A., Pechdee P., Thanchonnang C., La N., Rattanapitoon N.K., Arunsan P., Rattanapitoon S.K. (2023). Molecular detection of oral *Trichomonas tenax* in periodontal disease patients by polymerase chain reaction-based 18S rRNA gene. Trop. Biomed..

